# Influence of Sputtering Power on the Properties of Magnetron Sputtered Tin Selenide Films

**DOI:** 10.3390/ma17133132

**Published:** 2024-06-26

**Authors:** Krzysztof Mars, Mateusz Sałęga-Starzecki, Kinga M. Zawadzka, Elżbieta Godlewska

**Affiliations:** 1 Faculty of Materials Science and Ceramics, AGH University of Krakow, al. A. Mickiewicza 30, 30-059 Krakow, Poland; salegast@agh.edu.pl (M.S.-S.); godlewsk@agh.edu.pl (E.G.); 2 Faculty of Metals Engineering and Industrial Computer Science, AGH University of Krakow, al. A. Mickiewicza 30, 30-059 Krakow, Poland; kinga@agh.edu.pl

**Keywords:** tin selenide, magnetron sputtering, thermoelectric technology

## Abstract

The ecofriendly tin selenide (SnSe) is expected to find multiple applications in optoelectronic, photovoltaic, and thermoelectric systems. This work is focused on the thermoelectric properties of thin films. SnSe single crystals exhibit excellent thermoelectric properties, but it is not so in the case of polycrystalline bulk materials. The investigations were motivated by the fact that nanostructuring may lead to an improvement in thermoelectric efficiency, which is evaluated through a dimensionless figure of merit, ZT = S^2^ σ T/λ, where S is the Seebeck coefficient (V/K), σ is the electrical conductivity (S/m), λ is the thermal conductivity (W/mK), and T is the absolute temperature (K). The main objective of this work was to obtain SnSe films via magnetron sputtering of a single target. Instead of common radiofrequency (RF) magnetron sputtering with a high voltage alternating current (AC) power source, a modified direct current (DC) power supply was employed. This technique in the classical version is not suitable for sputtering targets with relatively low thermal and electrical conductivity, such as SnSe. The proposed solution enabled stable sputtering of this target without detrimental cracking and arcing and resulted in high-quality polycrystalline SnSe films with unprecedented high values of ZT equal to 0.5 at a relatively low temperature of 530 K. All parameters included in ZT were measured in one setup, i.e., Linseis Thin Film Analyzer (TFA). The SnSe films were deposited at sputtering powers of 120, 140, and 170 W. They had the same orthorhombic structure, as determined by X-ray diffraction (XRD), but the thickness and microstructure examined by scanning electron microscopy (SEM) were dependent on the sputtering power. It was demonstrated that thermoelectric efficiency improved with increasing sputtering power and stable values were attained after two heating–cooling cycles. This research additionally provides further insights into the DC sputtering process and opens up new possibilities for magnetron sputtering technology.

## 1. Introduction

Tin selenide (SnSe) has been drawing the attention of researchers since Zhao et al. [1] first reported the excellent thermoelectric properties of SnSe single crystals in a wide temperature range from 300 to 950 K. The parameters measured along the b axis changed in the following way: Seebeck coefficient from about 550 to 350 µV/K, electrical conductivity from 10 to almost 100 S/cm, thermal conductivity from 0.7 to 0.3 W/mK, which gave a thermoelectric figure of merit of 2.6 at 923 K. This high value was attributed to the transformation from a low-temperature orthorhombic to a cubic structure [2]. At room temperature, tin selenide has a layered structure with strong covalent bonds within each layer and weaker van der Waals bonds between the layers. This structure leads to anisotropy of properties, both in high- and low-temperature phases.

In view of the exceptionally good properties of tin selenide in single-crystal form, researchers started investigating its polycrystalline forms, which might be more durable and easier to produce. However, the undoped polycrystalline bulk materials had far worse properties than the single crystals [3].

One possible alternative was to obtain tin selenide in the form of a thin film, where additional effects might be activated and contribute to the enhancement of thermoelectric properties (phonon scattering or quantum confinement effects) [4,5]. The SnSe thin films were previously deposited by different chemical or physical methods, such as chemical vapour deposition (CVD), atomic layer deposition (ALD), electrodeposition (ED), deposition from a chemical bath, spray pyrolysis (SP), RF magnetron sputtering, hot-wall epitaxy (HWE), selenization of sputtered tin films, flash evaporation of SnSe compound [6,7,8,9,10,11,12,13,14,15]. Magnetron sputtering seems a particularly attractive technology as it is solvent-free, material-saving, scalable, and already widely used in various industries, including electronics, automotive, aerospace, and packaging [13,16,17]. Among the many variations of this process, pulsed DC sputtering of the SnSe target is rarely employed [16] because of its intrinsically low thermal and electrical conductivities and premature failure as a result of arcing and cracking. The arcing phenomena generate defects in the target and in the deposited film. In this work, an attempt was made to solve this problem using a suitable modification of the power supply. It was expected that the proposed solution would result in an extended lifetime of the target by enabling its uniform exploitation at high voltage without arcing. Special focus was directed at the effect of sputtering power and thermal history on the thermoelectric properties of the SnSe films.

## 2. Materials and Methods

**Figure 1:**
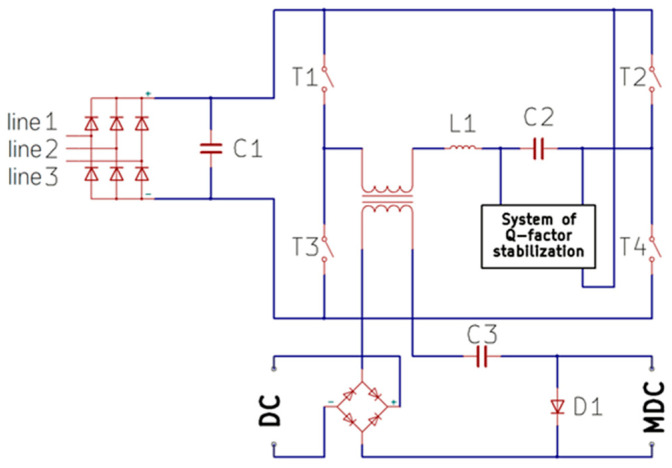
Scheme of DPS power supply: transistors are marked T1–T4, capacitors are marked C1–C3, and the coil is marked L1. The system can be operated either in DC (direct current) or MDC (modified direct current) mode.

All deposition processes were conducted at room temperature under argon (working gas) at about 1.5 × 10^−3^ mbar. A SnSe target with a diameter of 60 mm, thickness of 4 mm, and purity of 99.99% (Cathay Advanced Materials Limited, Guangdong, China) was installed in a planar magnetron gun supported by a 10 kW Dora Power System (DPS), Wroclaw, Poland, power supply. The substrates were parallel to the target and the target-to-substrate distance was 60 mm. Diverse substrates-(001) silicon wafers, glass plates, and microchips were used in each deposition process to receive a sufficient number of samples for the analyses of film topography, thickness, crystal structure, phase, and chemical composition, as well as thermoelectric properties using a Linseis Thin Film Analyzer (TFA), Selb, Germany [18,19]. The DPS power supply, shown in Figure 1, working in a full resonance mode with a series-connected energy receiver, contains a three-phase rectifier and a series resonant circuit powered by a full-bridge transistor system.

In this system, the energy stored in the capacitors is transferred to the receiver (magnetron gun) with a 90° phase shift through a switching system (MOSFET switches) and an LC resonance circuit. The voltage available at the output of the three-phase bridge, after smoothing by capacitors, is converted into a square waveform using a full bridge of four MOSFET transistors. The full bridge of transistor keys generates a square wave that powers the series resonant circuit. In the active (ON) state, transistor pairs T1–T4 or T2–T3 are alternately switched. In the passive (OFF) state, transistors T3–T4 are active. The transistor bridge operates in a four-step mode. In step 1, the first diagonal pair operates and then in step 2, the other one operates. In step 3, all transistors are turned off and then in step 4, one transistor from each pair connects to the negative terminal of the power supply, which enables the discharge of energy accumulated in the resonance circuit during the passive state (OFF). The amount of energy used for magnetron sputtering is controlled by the number of square pulses delivered to the modulated resonance circuit. The waveforms of voltage and current variations during the deposition of SnSe films at different sputtering powers are presented in Section 3.4: Analysis of electrical signals of the sputtering power source. This results in the generation of sinusoidal pulse groups dependent on the width of the modulating pulse. The modulation pulse repetition frequency varies between 1 Hz and 4 kHz and produces pulse groups ranging from single pulses to several hundred pulses. The switching frequency of the bridge circuit is adjusted to the resonant frequency of the system, derived from the LC parameters, ranging from 65 kHz to 75 kHz. Stabilization of the resonant circuit quality factor enables operation of the power supply either as a voltage source or a current source. In the case of a low-impedance receiver, when stable discharge is attained, the DPS power supply operates as a current source. In the case of a high-impedance receiver (when the magnetron is inactive), the DPS power supply limits the voltage, which may reach approximately 1 kV. The available MOSFET power transistors work best at frequencies up to 100 kHz. This limitation means that in order to obtain the desired power, the L/C ratio must be adequately adjusted so that the circuit can work in resonance. Pulse groups available in the DPS power supply output circuit can be unipolar (DC power supply output) or bipolar (MDC power supply output). The MDC stands for modified direct current power supply. The unipolar pulses are directly used to power the magnetron in a direct current (DC) mode, whereas the bipolar ones can be used to power the magnetron in reactive processes or can be converted into DC pulses using a half-wave rectifier containing a dedicated circuit (Plasma booster) composed of a diode and a capacitor, which partially inverts the signal and doubles the anode–cathode voltage. The output signal powering the magnetron gun is taken in parallel with the diode.

The SnSe films were deposited for 20 min, using the MDC power supply output. The sputtering power was 120, 140, or 170 W. The target was pre-sputtered for 2 min to remove impurities. The substrates were placed on a rotating table to secure the uniform thickness of the films. The base pressure in the vacuum chamber was about 5 × 10^−5^ mbar. The major deposition parameters are listed in Table 1.

Chemical composition and morphology of the SnSe films were analysed using scanning electron microscopy (FEI Nova NanoSEM, Sydney, NSW, Australia) combined with energy-dispersive X-ray spectrometry. Tofwerk FIB-SIMS was employed to examine microstructure and composition throughout film thickness. Small amounts of material were gradually removed by rastering an area of 20 µm^2^ with a focused ion beam (FIB). The liquid metal ion source was gallium. The ion current was 300 pA and the impact energy was 30 kV. Microstructural imaging was performed using probe Cs-corrected Titan Cubed G2 60–300 with the ChemiSTEM™ system (FEI). High-angle annular dark field (HAADF) detector was used for imaging in a high-resolution scanning transmission microscopy (HR-STEM) mode. The chemical composition was determined using energy-dispersive X-ray spectrometry (STEM-EDS). Samples for STEM studies were cut into thin lamellas using a Crossbeam 350 microscope (ZEISS, Oberkochen, Germany) equipped with a focused ion beam (FIB). The phase composition of the films was studied by Grazing-Incidence X-ray diffraction (GIXRD) using an Empyrean Panalytical diffractometer and CuKα1 radiation (λ = 0.1540598 nm) at an incidence angle of 0.5°. For incident beam optics, a Si-focusing mirror with both a 1/8° divergence slit and a Soller slit was employed. Thermoelectric properties of the SnSe films, including electrical conductivity, Seebeck coefficient, and thermal conductivity, were measured in the temperature range 300–530 K under a vacuum of 2 × 10^−5^ mbar using a Linseis Thin Film Analyser (TFA). Film thickness was measured with a Bruker DektakXT Stylus Profiler, Billerica, MA, USA. The end value was taken as an average of 5 measurements.

## 3. Results and Discussion

### 3.1. X-ray Diffraction

**Figure 2:**
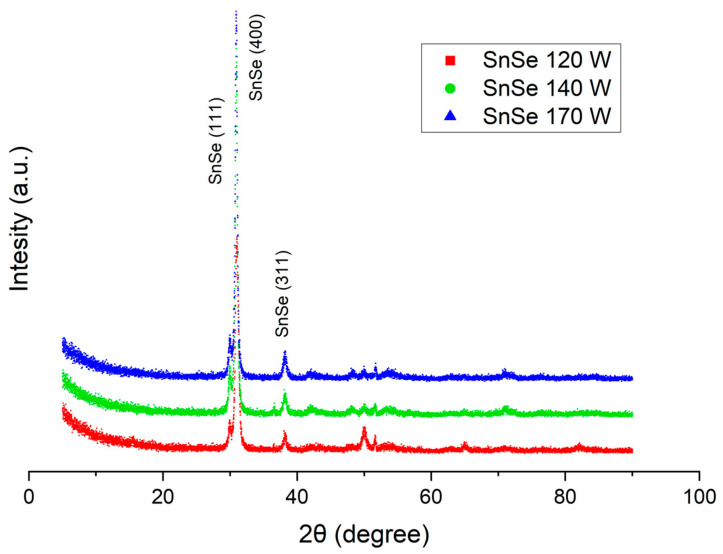
XRD patterns of the SnSe films received at different sputtering powers. Only the main reflections are labelled.

The X-ray diffraction patterns of the SnSe films, presented in Figure 2, are characteristic of the low-temperature orthorhombic structure (Pnma, space group 62), identified with JCPDS card no. 00-014-0159.

The major diffraction peaks found at about 30° and 39° are ascribed to the (111), (400), and (311) planes, respectively. With the increasing sputtering power, films grow in thickness and the corresponding peak intensities become higher. The evaluated unit cell parameters are in good agreement with the data reported in the literature [20]: a = 1.1490 nm, b = 0.4440 nm, c = 0.4135 nm. The diffraction patterns from the powdered SnSe target are also consistent with these data, which indicates that the composition and structure of the sputtered material are reproduced in the film.

### 3.2. Scanning Electron Microscopy

**Figure 3:**
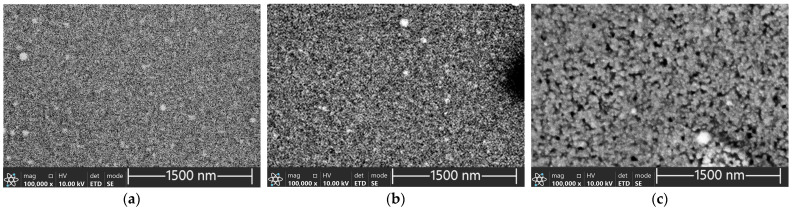
SEM micrographs of the SnSe films deposited on TFA microchips at sputtering powers of (**a**) 120 W, (**b**) 140 W, and (**c**) 170 W after measurement in TFA involving three consecutive heating–cooling cycles (top view).

The SEM micrographs of films received at sputtering powers of 120 W, 140 W, and 170 W are presented in Figure 3.

**Figure 4:**
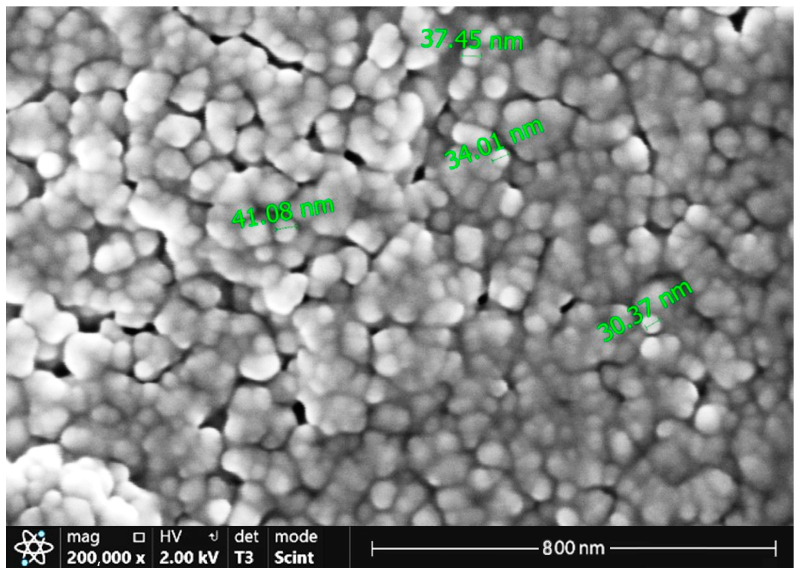
Surface of the film deposited at a sputtering power of 170 W after three heating–cooling cycles under a higher magnification (SEM micrograph).

The films were crystalline and with the increasing sputtering power the microstructure got coarser. At 170 W, granular crystallites with diameters ranging from about 30 to 40 nm were distinctly visible (Figure 4).

The phenomenon has been reported previously [21]. At constant sputtering times, thin films deposited at high sputtering power are thicker and have stronger crystallinity and better electrical conductivity compared with films deposited at low sputtering power. The high sputtering power favours nucleation and growth of nuclei in the form of 3D islands. This can be explained by the improved mobility of adatoms on the substrate surface. High power in the magnetron sputtering system provides energy to inert argon atoms, which in turn transfer sufficient kinetic energy to adatoms, enhancing their surface diffusion and momentum transfer to nucleation and growth of nuclei.

Based on EDS analysis, the chemical composition of the films was close to the SnSe stochiometric ratio; however, at the lowest sputtering power, some deviation toward selenium excess was recorded, as shown in Table 2. This result might be ascribed to the fact that the deposition process was less stable than in the case of higher sputtering power.

**Figure 5:**
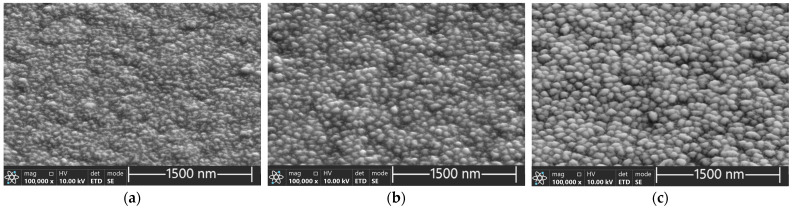
FIB-SEM micrographs of the subsurface area of the SnSe film deposited on a silicon wafer at a sputtering power of 170 W. Increasing frame numbers correspond to increasing distances from the surface: (**a**) frame 20; (**b**) frame 40, and (**c**) frame 50 (top view).

The FIB-SEM technique enabled the examination of film topography at different distances from the surface. As can be seen in Figure 5, stepwise removal of small portions of material from the surface of film deposited at a sputtering power of 170 W revealed systematic changes in the size of crystallites. The depth of analysis was defined by frame number. Close to the substrate (frame 50), the crystallites were densely packed and coarser than near the surface (frame 20).

**Figure 6:**
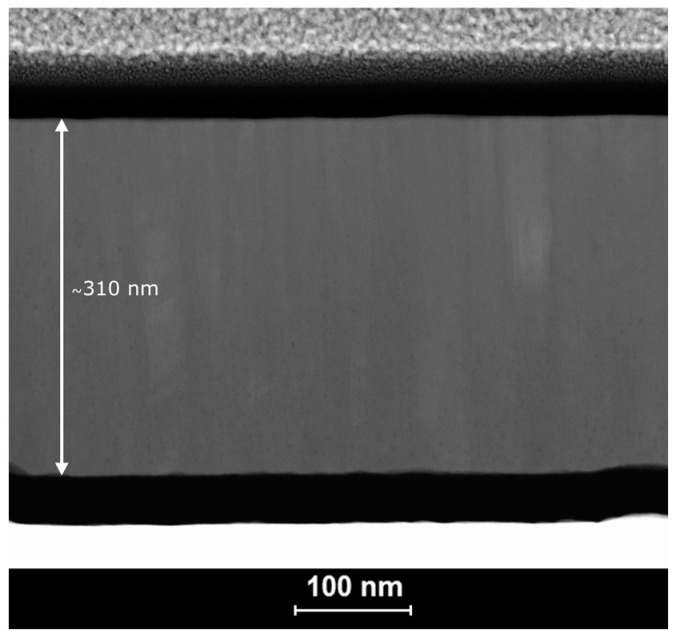
HAADF-STEM micrograph of a SnSe film deposited at a sputtering power of 170 W after three heating–cooling cycles in TFA. The layers visible on the micrograph from top to bottom are platinum, carbon, SnSe film (with marked thickness), carbon, and silicon on the microchip wafer.

TEM-EDS studies evidenced excellent quality of the SnSe films: uniform thickness and composition and lack of defects. An example of the HAADF-STEM micrograph presented in Figure 6 reveals densely packed columnar crystals growing in the direction perpendicular to the substrate. Their width was in good agreement with the crystallite diameters measured on the surface.

### 3.3. Thermoelectric Properties

Data, including electrical conductivity, σ, thermal conductivity, λ, and Seebeck coefficient, S, were recorded during three consecutive heating–cooling cycles to confirm the reproducibility of the measurements. As can be seen in Figures the curves corresponding to the second and third runs almost overlap. It was assumed, therefore, that after the third cycle, the films attained stability. The value of ZT = S^2^ σ T/λ was calculated for each of the investigated films. It is noteworthy that all parameters included in ZT could be measured on the same sample and in the same experimental setup (TFA), which ensures the credibility of the obtained results.

#### 3.3.1. Electrical Conductivity

**Figure 7:**
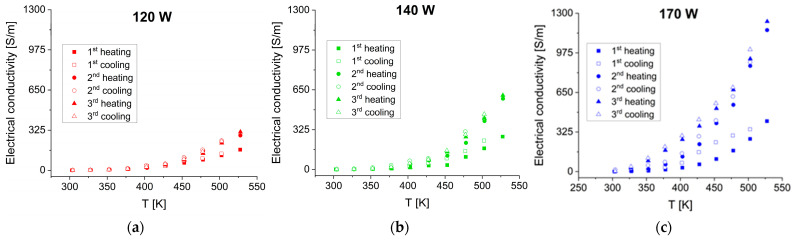
Temperature dependence of electrical conductivity for SnSe films obtained at sputtering powers of (**a**) 120 W, (**b**) 140 W, and (**c**) 170 W. The measurements were taken during three consecutive heating–cooling cycles in TFA.

Electrical conductivity was significantly affected by changes in temperature and sputtering power, as is visible in Figure 7. The rising trend indicates that the films had semiconducting properties.

**Figure 8:**
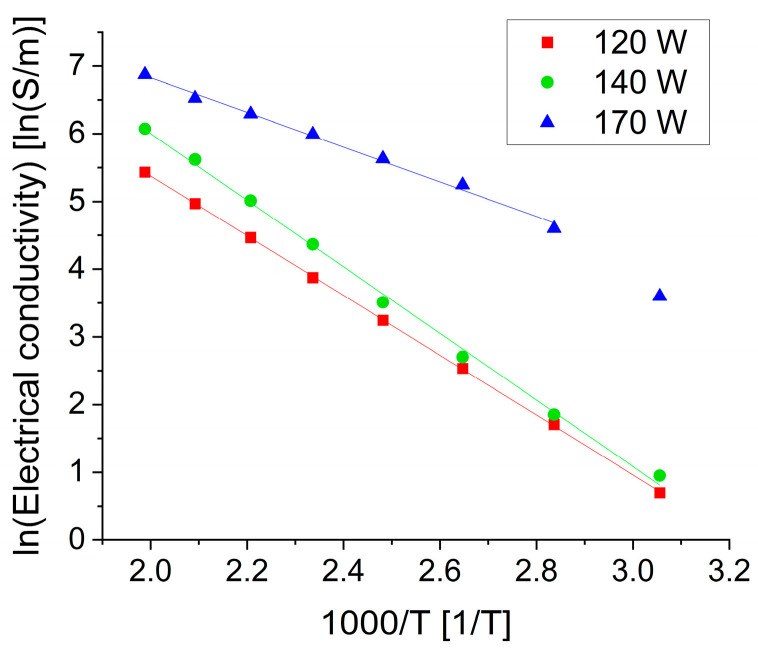
Temperature dependence of electrical conductivity in the Arrhenius plot of SnSe films obtained at sputtering powers of 120 W, 140 W, and 170 W. Only the average values from the third heating–cooling cycle in TFA were taken for this plot.

The maximum value of electrical conductivity is equal to 1232 S/m at 530 K and 170 W sputtering power is almost four times higher compared with 311 S/m at the same temperature and the lowest sputtering power of 120 W. The differences in electrical conductivity can be caused by electronic and morphological defects. Accordingly, at a similar concentration of charge carriers, the electrical conductivity may become lower because of scattering processes, which are more probable in thinner nanostructured films. A plausible explanation for the improvement of electrical conductivity in the second and third runs is that some ordering processes at the atomic level take place, which can be detected only indirectly through the measurements of some defect-sensitive properties. The temperature dependence of electrical conductivity in the Arrhenius plot (Figure 8) shows very similar slopes of the straight lines corresponding to films deposited at sputtering powers of 120 and 140 W. The line corresponding to the highest sputtering power is less inclined, i.e., the activation energy of electrical conductivity is lower starting from about 350 K.

#### 3.3.2. Seebeck Coefficient

**Figure 9:**
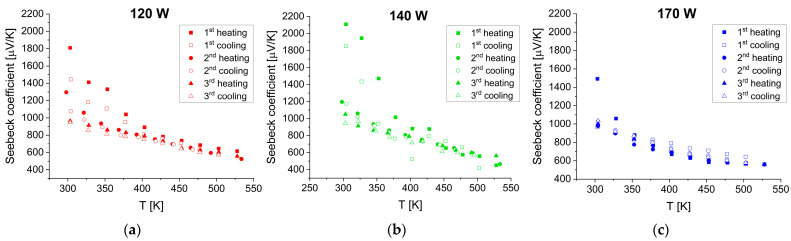
Seebeck coefficient as a function of temperature in the SnSe films deposited at sputtering powers of (**a**) 120 W, (**b**) 140 W, and (**c**) 170 W. The measurements were taken during three consecutive heating–cooling cycles in TFA.

Temperature variations in the Seebeck coefficient for SnSe films deposited at different sputtering powers are shown in Figure 9.

In each case, the films have similar characteristics. The measured values are positive and decrease from about 1000 μV/K at 300 K to about 560 μV/K at 530 K in the third heating–cooling cycle. The Seebeck coefficient is an intrinsic material property that measures the thermoelectric voltage induced in response to a temperature difference across the material and is directly related to the density of states. The sign indicates the type of major charge carriers—the positive sign denotes electron holes. The decreasing trend of the temperature dependence of the Seebeck coefficient can be associated with increasing concentration of charge carriers.

#### 3.3.3. Thermal Conductivity

**Figure 10:**
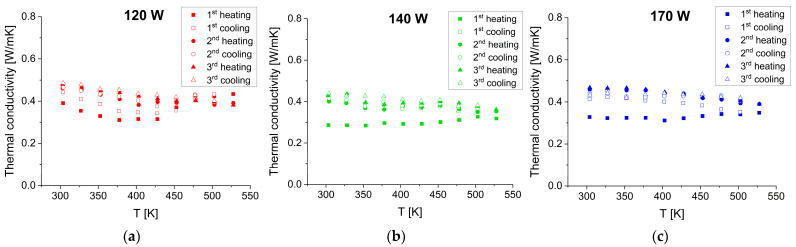
Thermal conductivity as a function of temperature for the SnSe films deposited at sputtering powers of (**a**) 120 W, (**b**) 140 W, and (**c**) 170 W. The measurements were taken during three consecutive heating–cooling cycles in TFA.

Thermal conductivity is a very important parameter in thermoelectric systems as it determines the efficiency of heat flow through materials. Total thermal conductivity consists of contributions from both electron and phonon transport, i.e., electron and lattice thermal conductivity. The latter is known as a dominating mechanism of heat conduction in semiconductors at temperatures close to room temperature. The temperature dependence of thermal conductivity for the SnSe films is shown in Figure 10.

Also, in this case, stabilization takes place after the second heating–cooling cycle. The values of thermal conductivity drop from about 0.5 to 0.3 as the temperature increases from 300 to 530 K, independently of the applied sputtering power.

#### 3.3.4. Thermoelectric Figure of Merit (ZT)

**Figure 11:**
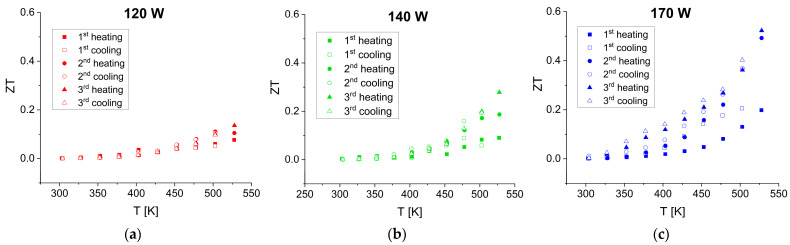
Dependence of ZT on temperature for the SnSe films deposited at sputtering powers of (**a**) 120 W, (**b**) 140 W, and (**c**) 170 W over three consecutive heating–cooling cycles in TFA.

The calculated ZT parameter is plotted against temperature in Figure 11.

**Figure 12:**
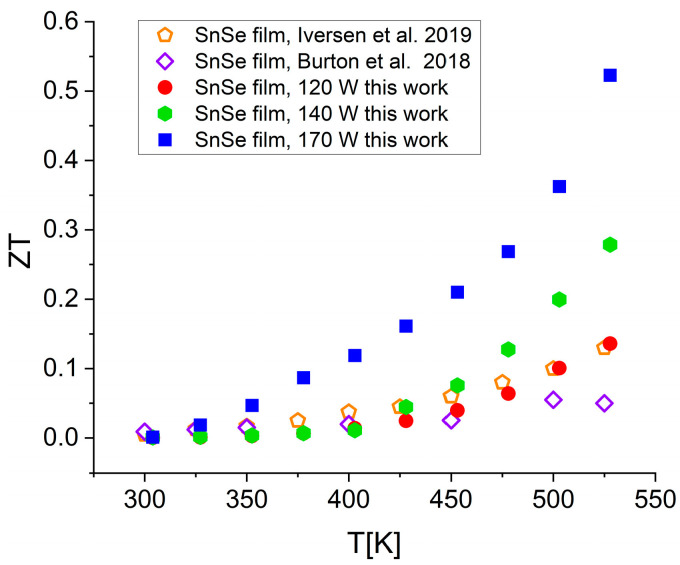
Comparison of the temperature dependence of ZT of the SnSe thin films obtained in this work with the data reported in [16,22].

As can be seen, ZT continuously increases with temperature and sputtering power. For all the films, the values at room temperature are very low but change markedly at higher temperatures and become repeatable starting from the second cycle. At 530 K, ZT reaches 0.13, 0.28, and 0.53 for the films deposited at sputtering powers of 120 W, 140 W, and 170 W, respectively. The thermoelectric efficiency of the SnSe films deposited in this work is compared with the previously reported data in Figure 12. The references [16,22] were chosen because the authors used SnSe films with relatively high ZT values.

These, however, appear lower than or similar to the ZT of SnSe films deposited in this work at a sputtering power of 120 W. At 140 W or 170 W, the thermoelectric figure of merit becomes significantly higher starting from about 450 K, exceeding the values reported by Iversen et al. [16] by more than two times. It is worth emphasizing that here the measurements were taken over a temperature range where bulk SnSe samples do not exhibit good thermoelectric properties [23].

The films synthesized by Iversen et al. [16] were sputtered from a single target and annealed for 1 h at 700 K. They were dense, crack-free, consisted of tightly packed grains, 100–300 nm in diameter, and had a thickness of about 650 nm. Their electrical properties—conductivity and Seebeck coefficient—were measured on a ZEM-3 setup but thermal conductivity, necessary for calculating ZT, was taken from bulk SnSe [24]. The SnSe films reported by Burton et al. [22] were deposited via thermal evaporation. They consisted of nanosheets or nanoplatelets growing in the direction perpendicular to the substrate and had a total thickness of about 1000 nm. The researchers measured thermal conductivity using Linseis TFA and used ZEM-3 for measuring electrical conductivity and Seebeck coefficient. In Table 3, we outline the characteristic parameters of SnSe in different forms at 530 K: films having different thicknesses, bulk monocrystals, and polycrystals.

The Seebeck coefficient of the best film obtained in this work, 559 μV/K at 530 K, is close to the 500 μV/K reported by Iversen et al. [16] but much higher than the 210 μV/K reported by Burton et al. [22]. The thermal conductivity of 0.39 W/mK is lower than the 0.65 W/mK taken for calculations by Iversen et al. [16], and much higher than the 0.12 W/mK reported by Burton et al. [22]. The corresponding values of electrical conductivity are 1232, 589, and 255 S/m. Unquestionably, the SnSe 170 W film with a power factor of 3.85 µW/cmK^2^ outperforms all the other materials listed in Table 3, including the monocrystal. There might be multiple reasons for the differences: film thickness, integrity, microstructure, and composition. All of these are dependent on deposition conditions. In our work, film thickness was dependent on sputtering power only because deposition time was always the same (20 min). It is known that the thickness of films may have a significant effect on their thermoelectric properties [22,26,27,28], and especially on electrical conductivity: the thinner the films, the stronger the carrier scattering. This tendency can be noticed also in the series of SnSe films obtained in this work. Other films quoted in Table 3 were much thicker, but their electrical properties did not change in the same direction because their microstructures were not comparable.

In this work, we demonstrated that an increase in sputtering power led to better thermoelectric properties. On the one hand, this effect could be ascribed to increasing the thickness of the films and on the other hand, to better compactness and structuring resulting from the amount of energy released on the surface of the target. The maximum sputtering power, 170 W, was actually at the upper limit of process stability. In Table 4, we present the deposition parameters for the films listed in Table 3.

It can be seen that the power density on the surface of the target at a sputtering power of 170 W was three times higher than that achieved in RF sputtering by Iversen et al. [16]. Also, the deposition rate was higher by about 50%. This may explain the differences in the microstructure of the SnSe films.

### 3.4. Analysis of Electrical Signals of the Sputtering Power Source

**Figure 13:**
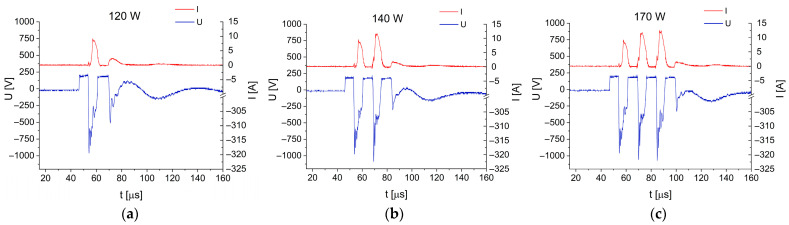
Voltage and current characteristics during the deposition of SnSe films at increasing sputtering powers: (**a**) 120 W (**b**) 140 W; (**c**) 170 W.

The magnetron gun was powered by a pulsed power supply in which the alternating sinusoidal signal was converted using a half-wave voltage multiplier based on capacitor C3 and diode D1. In order to better understand the operating conditions of the modified experimental setup, we present here variations of current and voltage between anode and cathode with time during the deposition process at sputtering powers of 120 W, 140 W, and 170 W (Figure 13).

The form of oscillograms depends on the structural components of the pulsed power supply (L1, C2, C3) and on the process conditions, such as the type and pressure of the working gas, the type of target, and the geometry of the sputtering gun. In our system, capacitor C3 is charged to a voltage twice as high as that available in the resonant circuit, which reaches about 1300 V at the magnetron sputtering gun. This voltage is sufficiently high to activate glow discharge. During the sputtering process, the power supply operates as a current source, stabilizing the sputtering current. Pulse suppression is related to the discharge of energy stored in capacitor C3. The anode–cathode voltage waveform reflects the plasma response to the excitation generated by the current signal from the power supply. The distortion of the anode–cathode voltage waveforms shown in Figure 13 is caused by plasma oscillations associated with the ionization of various atoms present in the working gas (Ar) upon sputtering of the SnSe target. After the pulse is extinguished, the plasma is turned off (I = 0 A) and the system can start a high voltage upon the next pulse. Between pulses, the voltage at the power supply output is 0 V. Positive voltage values measured between the anode and cathode can result from charging of the target, with positive argon ions reaching the target surface with a delay.

The number of pulses and the amplitude change of the last pulse in the group enables smooth power control at the magnetron gun. Plasma turning-off between the pulses in the group effectively limits the number of arc discharges, which may damage the target and generate defects in the deposited film. This issue becomes of particular importance in the case of the SnSe target, which is characterized by low electrical and thermal conductivities. The modified power supply system permits stable sputtering within the medium frequency range and power density on the target sufficient to secure a high deposition rate.

## 4. Conclusions

The MDC power supply used in this work brought about significant improvements in the exploitation of the high-impedance SnSe target, enabling uniform and controlled sputtering without arcing. The SnSe films—compact, uniform in thickness and composition—were deposited at a relatively high rate. Film thickness progressively increased with the sputtering power and at 170 W, reached 300 nm within 20 min. The very good quality of SnSe films can be attributed to the electrical parameters of the glow discharge plasma in magnetron sputtering. With the MDC power supply, the anode–cathode voltage could reach more than 1000 V, which apparently translates into the high energy of ions travelling from the target toward the substrate (this energy changes with voltage squared).

In the investigated temperature range, from 300 to 530 K, limited by the Linseis TFA specifications, the SnSe 170 W film outperformed all other reported SnSe films and bulk samples, including monocrystals, in terms of thermoelectric properties. Its dimensionless figure of merit increased with temperature and at 530 K reached an unprecedented value of 0.53 and a power factor of 3.85 µW/cmK^2^. This is a very promising result, encouraging further studies. Among the scientifically and technologically interesting issues are the properties of glow discharge plasma, identification of electronic and ionic currents, dependence of film thickness on time at a constant sputtering power, dependence of thermoelectric properties on film thickness, and identification of structural details of the films by electron diffraction and high-resolution imaging techniques.

## Figures and Tables

**Table 1:** Deposition parameters used in this work.

Sample Name	Sputtering Power (W)	Film Thickness (nm)	Time (min)	Argon Pressure (mbar)
SnSe 120 W	120	133	20	1.5 × 10^−3^
SnSe 140 W	140	220	20	1.5 × 10^−3^
SnSe 170 W	170	310	20	1.5 × 10^−3^

**Table 2:** Chemical composition of the SnSe films determined by EDS.

Sample Name	Se (at. %)	Sn (at. %)
SnSe 120 W	53.6 ± 7.2	46.4 ± 6.5
SnSe 140 W	50.1 ± 6.8	49.9 ± 4.2
SnSe 170 W	49.6 ± 6.6	50.4 ± 3.8

**Table 3:** Thermoelectric properties of SnSe in different forms at about 530 K.

Sample Name	Film Thickness (nm)	ZT (-)	PF (µW/cmK^2^)	Electrical Conductivity (S/m)	Seebeck Coefficient (μV/K)	Thermal Conductivity (W/mK)
SnSe 120 W	133	0.13	0.98	331	559	0.38
SnSe 140 W	220	0.28	1.91	605	562	0.36
SnSe 170 W	310	0.52	3.85	1232	559	0.39
Iversen et al. [16]	650	0.13	1.45	589	500	0.65
Burton et al. [22]	~1000	0.05	0.11	255	210	0.12
Monocrystalline (b axis) [1]	bulk	0.18	1.6	512	566	0.47
Polycrystalline [25]	bulk	0.03	0.42	227	433	0.80

**Table 4:** Deposition parameters for the discussed SnSe films.

Sample Name	Film Thickness (nm)	Power Density (W/cm^2^)	Deposition Rate (nm/min)	Type of Power Supply
SnSe 120 W	133	4.3	6.65	MDC
SnSe 140 W	220	5.0	11	MDC
SnSe 170 W	310	6.0	15.5	MDC
Iversen et al. [16]	650	2.0	10.83	RF
Burton et al. [22]	~1000	-	-	-

## Data Availability

The raw data supporting the conclusions of this article will be made available by the authors on request.
